# Myricetin 3-*O*-β-D-Galactopyranoside Exhibits Potential Anti-Osteoporotic Properties in Human Bone Marrow-Derived Mesenchymal Stromal Cells via Stimulation of Osteoblastogenesis and Suppression of Adipogenesis

**DOI:** 10.3390/cells10102690

**Published:** 2021-10-08

**Authors:** Fatih Karadeniz, Jung Hwan Oh, Hyun Jin Jo, Youngwan Seo, Chang-Suk Kong

**Affiliations:** 1 Marine Biotechnology Center for Pharmaceuticals and Foods, College of Medical and Life Sciences, Silla University, Busan 46958, Korea; karadenizf@outlook.com (F.K.); wjdghks0171@naver.com (J.H.O.); 2 Department of Food and Nutrition, College of Medical and Life Sciences, Silla University, Busan 46958, Korea; genie970909@naver.com; 3 Division of Marine Bioscience, Korea Maritime and Ocean University, Busan 49112, Korea; ywseo@kmou.ac.kr

**Keywords:** adipogenesis, MSC, myricetin 3-*O*-β-D-galactopyranoside, osteoblastogenesis, Wnt/BMP

## Abstract

Natural bioactive substances are promising lead compounds with beneficial effects on various health problems including osteoporosis. In this context, the goal of this study was to investigate the effect of myricetin 3-*O*-β-D-galactopyranoside (M3G), a glycoside of a known bioactive phytochemical myricetin, on bone formation via osteogenic differentiation of human bone marrow-derived mesenchymal stromal cells (hBM-MSCs). The hBM-MSCs were induced to differentiate into osteoblasts and adipocytes in the presence or absence of M3G and the differentiation markers were analyzed. Osteoblastogenesis-induced cells treated with M3G exhibited stimulated differentiation markers: cell proliferation, alkaline phosphatase (ALP) activity, and extracellular mineralization. In terms of intracellular signaling behind the stimulatory effect of M3G, the expression of RUNX2 and osteopontin transcription factors were upregulated. It has been shown that M3G treatment increased the activation of Wnt and BMP as a suggested mechanism of action for its effect. On the other hand, M3G treatment during adipogenesis-inducement of hBM-MSCs hindered the adipogenic differentiation shown as decreased lipid accumulation and expression of PPARγ, SREBP1c, and C/EBPα, adipogenic transcription factors. In conclusion, M3G treatment stimulated osteoblast differentiation and inhibited adipocyte differentiation in induced hBM-MSCs. Osteoblast formation was stimulated via Wnt/BMP and adipogenesis was inhibited via the PPARγ pathway. This study provided necessary data for further studies to utilize the therapeutic potential of M3G against osteoporosis via regulation of bone marrow stromal cell differentiation.

## 1. Introduction

During osteoporosis progression, it was shown that the differential tendencies of bone marrow mesenchymal stromal cells (MSCs) shift towards adipocytes rather than osteoblasts which lead to a bone formation imbalance [1]. This imbalance was credited as the reason behind the porous and fragile bone structure seen in patients with osteoporosis. Treating osteoporosis was carried out with a broad range of approaches that target different steps of osteoporotic complications. To date, most of these treatments aimed to increase bone formation employing different types of drug classes such as bisphosphonates, parathyroid hormone, and selective estrogen receptor modulators. Studies have provided evidence that the differential regulation of bone marrow multipotent cells is directly linked with the bone structure and development [1,2]. Considering the correlation between adipogenesis and osteoblastogenesis mechanisms of the MSCs, it was hypothesized that tilting the MSC differentiation towards osteoblasts could result in beneficial effects in osteoporotic progression by decreasing adipose tissue and increasing bone formation. Regulation of MSC differentiation is handled by different signaling pathways which, however, have antagonistic relations when it comes to adipogenesis and osteoblastogenesis [3]. Inducing MSCs into osteoblastogenesis occurs via activation of osteogenic signaling which at the same time suppresses adipogenic stimuli and vice versa. The main pathways that induce adipogenesis and osteoblastogenesis and in this kind of intertwined relation are peroxisome proliferator-activated receptor γ (PPARγ) and Wnt/β-catenin signaling, respectively [4]. Inducing MSCs to differentiate into adipocytes through PPARγ activation inhibits the bone morphogenetic protein (BMP)-induced osteoblastogenesis while activation of Wnt/β-catenin for osteogenic differentiation concurrently hinders adipogenesis by suppressing PPARγ transcriptional activities. 

**Figure 1:**
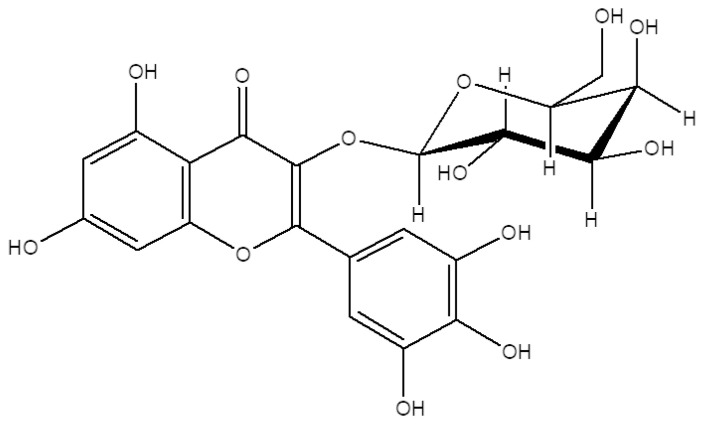
Chemical structure of M3G.

Plant-based bioactive substances make up most of the reported natural products with promising beneficial effects. Among these substances, the notable ones that cover the biggest shares were flavonoids, coumarins, and terpenes possessing therapeutic properties for the symptoms of global diseases such as obesity, diabetes, cardiovascular diseases, and osteoporosis [5,6]. Flavonoids are very common polyphenol secondary metabolites found in plants and fungus and can be found in various commercial products on the market with medical, nutritional, and cosmeceutical applications [7]. Expectedly, several of these flavonoids were reported to possess anti-adipogenic or pro-osteogenic properties with different action mechanisms and therapeutical targets [8,9]. Myricetin is a flavonoid from the flavonol sub-class and one of the most common flavonoids in the daily human diet. Myricetin can be found in various plant species and is known for its nutraceutical value [10]. Similar to other bioactive flavonoids, myricetin is a good free radical scavenger and its derivatives exert different physiological benefits [11]. Reported nutraceutical properties of myricetin derivatives vary from reducing cardiovascular risks to anti-tumor, anti-diabetic and anti-aging activities [12,13,14]. In this context, the goal of the present study is to analyze the effect of a myricetin glycoside derivative, myricetin-3-O-β-D-galactopyranoside (M3G) (Figure 1), on the differentiation of human bone marrow-derived mesenchymal stromal cells (hBM-MSCs) into adipocytes and osteoblasts in order to provide insights towards its anti-osteoporotic potential. The results of the current study are expected to provide data for further studies to utilize M3G as a nutraceutical with MSC differentiation regulatory effects that can be used against metabolic disorders such as obesity and osteoporosis.

## 2. Materials and Methods 

### 2.1. M3G Isolation and Characterization

M3G was isolated from *Limonium tetragonum* methanolic crude extracts. The isolation and characterization of M3G was carried out as previously described [15]. Briefly, ^1^H and ^13^C NMR spectra were evaluated and compared with published literature for the identification of M3G.

### 2.2. Cell Culture and Differentiation

The current study used human bone marrow-derived mesenchymal stromal cells (hBM-MSCs) as in vitro models to analyze MSC differentiation into adipocytes and osteoblast. Cells were purchased from PromoCell (C-12974, Heidelberg, Germany). For the experiments, cells at passage numbers between 3 and 6 were cultured in 6-well plates (1 × 10^6^ cells/well) unless otherwise noted. Prior to differentiation, cells were grown to confluency during which they were fed mesenchymal stem cell growth medium (C-28009, PromoCell). Following confluency, hBM-MSCs were induced to differentiate into adipocytes or osteoblasts by swapping the culture medium with mesenchymal stem cell adipogenic differentiation medium 2 (C-28016, PromoCell) or mesenchymal stem cell osteogenic differentiation medium (C-28013, PromoCell), respectively. Cells introduced to differentiation media were incubated for 10 days, swapping the differentiation medium with fresh media every third day. Treatment with M3G was carried out along with initial differentiation inducement only. Incubation of hBM-MSCs before and during the differentiation was in controlled incubators set to 37 °C temperature and 5% CO_2_ level. 

### 2.3. Cell Viability Assay

Any cytotoxic effect that M3G might have on hBM-MSCs was evaluated with standard 3-(4,5-dimethylthiazol-2-yl)-2,5-diphenyltetrazolium bromide (MTT) assay [15]. Briefly, hBM-MSCs in 96-well plates (seeded at 1 × 10^3^ cell/well density) were incubated for 24 h prior to the assay. After 24 h, cells were treated with varying doses of M3G, introduced with fresh culture medium. Treatment lasted for 72 h, after which the wells were aspirated and added 100 μL of MTT reagent (1 mg/mL in culture medium) and incubated for 4 more hours. Next, 100 mL dimethyl sulfoxide (DMSO) was added to each well to stop the reaction and the absorbance values were measured at 540 nm. Cell viability was quantified as a relative percentage compared to M3G untreated control group absorbance values after the normalization with the blank group which only contained the reagents but not the cells.

### 2.4. Staining of Lipid Droplets in Adipocytes by Oil Red O Staining

As a marker of successful adipocyte differentiation and maturation, intracellular lipid accumulation was observed in adipo-induced hBM-MSCs at day 10 differentiation. Observation and quantification of the lipid accumulation of adipocytes were carried out with Oil Red O staining of the intracellular lipid droplets. Briefly, hBM-MSCs were induced to differentiate into adipocytes as described above. At day 10 of differentiation, the wells were aspirated, and the adipocytes were fixed on wells for 1 h by the addition of 10% fresh formaldehyde in phosphate-buffered saline (PBS) (*v*/*v*). Fixed cells were stained with 1 mL 0.5% Oil Red O staining solution (m/v in 60% isopropanol) in each well. Staining was carried out for 1 h in incubators. Unbound Oil Red O stain was washed with PBS and the stained lipid droplets were observed under a light microscope (Olympus, Tokyo, Japan). For the quantification of the stain, lipid-bound Oil Red O was eluted from wells by addition of 100% isopropanol to the wells. The eluted stain was then quantified via measuring absorbance values at 500 nm. Lipid accumulation was given as a relative percentage of Oil Red O stain eluted compared to M3G untreated control adipocytes. Absorbance values were normalized against blank wells that contained all the reagents but not the cells.

### 2.5. mRNA Expression Analysis

The hBM-MSCs differentiated into adipocytes or osteoblasts were analyzed for the mRNA expressions of adipogenic and osteogenic marker genes. At day 10 of differentiation, total RNA was extracted from non-differentiated and differentiated (M3G-treated and non-treated) hBM-MSC groups by following the given protocols of AccuPrep Universal RNA Extraction Kit (BioNeer, Daejeon, Korea). The cDNA was synthesized from extracted RNA (2 μg) using Cell Script All-in-One cDNA Synthesis Master Mix (CellSafe, Yongin, Korea) according to manufacturer’s instructions and following steps: 42 °C for 60 min and 72 °C for 5 min. Subsequently, polymerase chain reaction (PCR) analysis was carried out with Luna^®^ Universal qPCR Mix (New England Biolabs, Ipswich, MA, USA) in TP800 Thermal Cycler Dice™ Real-Time System (Takara Bio, Ohtsu, Japan). The amplification values were plotted as fold change compared to the differentiated but not M3G treated group. All amplification values were normalized against β-actin as the reference gene. Visualization of the PCR bands was carried out with electrophoresis (30 min at 100 V on a 1.5% agarose gel) of final products and subsequent ethidium bromide (1 mg/mL) staining. Stained gels were pictured with CAS-400SM Davinch-Chemi Imager™ (Davinch-K, Seoul, Korea).

### 2.6. Protein Level Analysis 

The protein levels of adipogenic and osteogenic markers were analyzed in differentiated hBM-MSCs via Western blotting. At day 10 differentiation, total protein was extracted from cell lysates obtained by addition of 1 mL RIPA buffer and pipetting. The supernatants of the cell lysates after centrifugation (13,000× *g*, 4 °C, 15 min) were used for Western blotting after assessing their total protein content with the BCA protein assay kit (Thermo Fisher Scientific, Waltham, MA, USA). Furthermore, nuclear protein content was extracted from total protein using the NE-PER^TM^ Nuclear Extraction Kit (#78835; Thermo Fisher Scientific). For the blotting, the same amount of protein from total or nuclear extracts of test groups (20 μg) was separated by sodium dodecyl-sulfate polyacrylamide gel electrophoresis (SDS-PAGE) (4% stacking and 10% separating gels). The transfer of proteins from gels to membranes was carried out with traditional wet transfer protocols for Western blotting using polyvinylidene fluoride membrane (Amersham Bioscience, Westborough, MA, USA). Transfer to membranes was followed by blocking the membranes for 4 h in 5% skim milk (*v*/*v*) in TBS-T buffer for 4 h. Then, the proteins were incubated overnight at 4 °C with primary antibodies diluted as suggested by the manufacturer in 1X TBS-T. The primary antibodies used in this study were given in a previous report [16]. Following the primary antibody hybridization, membranes were treated for 2 h with horseradish peroxidase-conjugated secondary antibodies specific to the primary antibody organism. Visualization of the protein bands was carried out by staining membranes with an enhanced chemiluminescent (ECL) kit (Amersham Bioscience) following the manufacturer’s protocol. The protein bands were pictured with CAS-400SM Davinch-Chemi imager (Davinch-K).

### 2.7. Cellular Alkaline Phosphatase (ALP) Activity

The activity of ALP obtained from differentiated hBM-MSC osteoblasts was evaluated with a commercial kit. At day 7 of differentiation, osteo-induced hBM-MSCs were lysed with the addition of 0.1% Triton X-100 and 25 mM carbonate buffer and pipetting. The supernatants obtained from cell lysates after centrifugation (12,000× *g*, 4 °C, 15 min) were first analyzed for their total protein content by the Bradford protein determination method. Next, the ALP activity in the same amount of protein from each test group was calculated with the Alkaline Phosphatase Activity Colorimetric Assay Kit (K412-500; BioVision, Hannover, Germany) following the producer’s protocol. The conversion rate of pNP by ALP was plotted after normalization to the total protein amount of cell samples.

### 2.8. Alizarin Red Staining

As a marker of osteoblast maturation, extracellular mineralization was analyzed by staining calcified nodules of differentiated hBM-MSC osteoblasts with Alizarin Red staining. At the day 10 of differentiation, hBM-MSC osteoblasts were fixed by swapping culture medium with 4 °C 70% ethanol. Following 1 h of incubation, ethanol was removed from wells and fixed osteoblasts were washed with distilled water. Two percent of Alizarin Red staining solution (m/v) with a pH of 4.2 was then added to the wells for 10 min of staining. Following staining, the solution was aspirated from wells and the dyed cells were washed with distilled water. Stained calcifications were pictured with an Olympus microscope (Tokyo, Japan) fitted with a camera. In order to quantify the staining, the Alizarin Red dye was eluted from stained cells with 10% (*m/v*) cetylpyridinium chloride in 10 mM sodium phosphate buffer (Sigma-Aldrich, St. Louis, MO, USA). Eluted dyes were measured for their absorbance at 560 nm using a Multiskan GO microplate reader (Tecan Austria GmbH, Grodig, Austria). Values were normalized against the blank group that contained only elution solution and plotted as a relative percentage of differentiated but not M3G-treated hBM-MSCs.

### 2.9. Immunohistochemical Staining

Marker proteins for adipocytes and osteoblasts in differentiated hBM-MSCs were visualized with immunofluorescence staining of the BMP2, and RUNX2 for osteoblasts and PPARγ, and perilipin-1 for adipocytes. The hBM-MSCs were cultured and differentiated as stated earlier except that the immunohistochemical staining wells were fitted with glass coverslips prior to cell seeding. The rest of the differentiation was carried out in the same manner as previous assays. At day 10 of differentiation, differentiated hBM-MSCs were fixed on coverslips and hybridized with antibodies conjugated with Alexa Fluor 488 (A-11008; Thermo Fisher Scientific) against perilipin-1 (ab3526; Abcam, Cambridge, UK), PPARγ (ab9256; Abcam), BMP2, and RUNX2. In the case of nuclei staining as a reference, the ProLong Gold Antifade reagent with DAPI (#8961; Cell Signaling Technology, Danvers, MA, USA) was used. The hBM-MSCs were fixed and stained by using the commercial solutions and protocols of the Immunofluorescence Application Solutions Kit (#12727; Cell Signaling Technology).

### 2.10. Statistical Analysis

Where applicable, all data were given as the average of three independent experiments (*n* = 3) (run in triplicate except Western blotting) ± SD unless otherwise described. Data groups were subjected to one-way analysis of variance (ANOVA) with post hoc Duncan’s multiple range test using SAS software (SAS v9.1, SAS Institute, Cary, NC, USA) and the minimum significant statistical difference was defined at *p* < 0.05 level.

## 3. Results

### 3.1. Proliferation, ALP Activity and Extracellular Mineralization of Osteo-Induced hBM-MSCs

**Figure 2:**
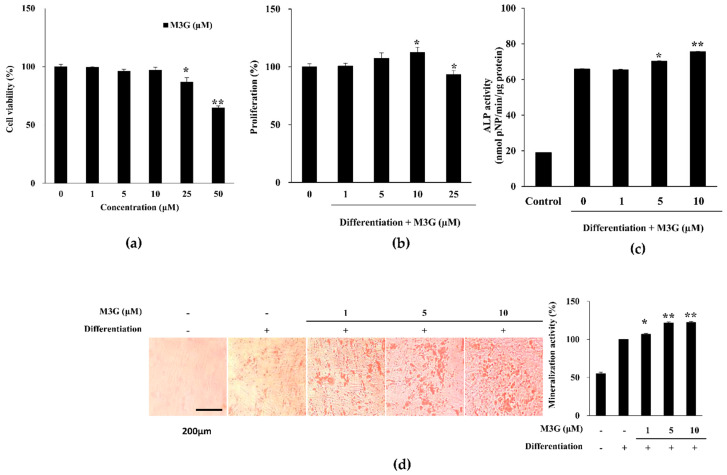
Effect of M3G on the osteoblastogenic differentiation of hBM-MSCs. Effect of M3G on the (**a**) viability of non-differentiated hBM-MSCs and (**b**) proliferation of osteo-induced hBM-MSCs. Viable cell amount was measured by quantification of MTT dye removed from cells at day 3 of differentiation. Proliferation was given as relative viable cell amount (%) of untreated osteo-induced control. (**c**) Effect of M3G on the activity of cellular ALP. Cellular ALP activity of osteo-induced hBM-MSCs was measured with a spectrophotometric enzymatic activity assay at day 7 of differentiation. M3G was present in the first 3 days of differentiation only. Control: Non-differentiated untreated cells in culture medium. (**d**) Effect of M3G on the extracellular mineralization of osteo-induced hBM-MSCs. Extracellular mineralization was measured by Alizarin Red staining and quantified by the absorbance values of the retained dye at day 10 of differentiation. Mineralization was given as a relative percentage of untreated osteo-induced hBM-MSCs. M3G was treated in the first 3 days of differentiation only. * *p* < 0.05, ** *p* < 0.01 vs. differentiated untreated group.

Treatment with M3G did not result in any viability loss in hBM-MSCs following 72 h treatment up to a concentration of 10 μM (Figure 2a). Starting from 25 μM, cell viability was observed to drop. On the other hand, hBM-MSCs treated with M3G with the initial osteogenic differentiation media (3 days) exerted increased viable cell amount in a dose-dependent manner until 10 μM (Figure 2b). However, at 25 μM concentration, the M3G-treated group exhibited a loss of viability compared to the untreated differentiated group. Therefore, the following studies were conducted using an M3G dose of up to 10 μM which was deemed safe.

ALP activity of the differentiated hBM-MSCs was analyzed at day 7 of differentiation. ALP activity significantly increased by the osteoblast differentiation from 18.98 U/mL of non-differentiated hBM-MSCs to 65.88 U/mL. At concentrations of 5 and 10 μM, M3G treatment significantly stimulated the ALP activity to the levels of 70.28 U/mL and 75.56 U/mL, respectively, compared to untreated hBM-MSC osteoblasts (Figure 2c). The level of ALP activity stimulation was expectedly observed in mineralization as well. M3G treatment dose-dependently elevated the extracellular calcification in hBM-MSC osteoblasts (Figure 2d). At the highest concentration treated (10 μM), Alizarin Red stained areas were 22.30% higher compared to untreated hBM-MSC osteoblasts. 

### 3.2. Osteoblast Marker Gene and Protein Expression

**Figure 3:**
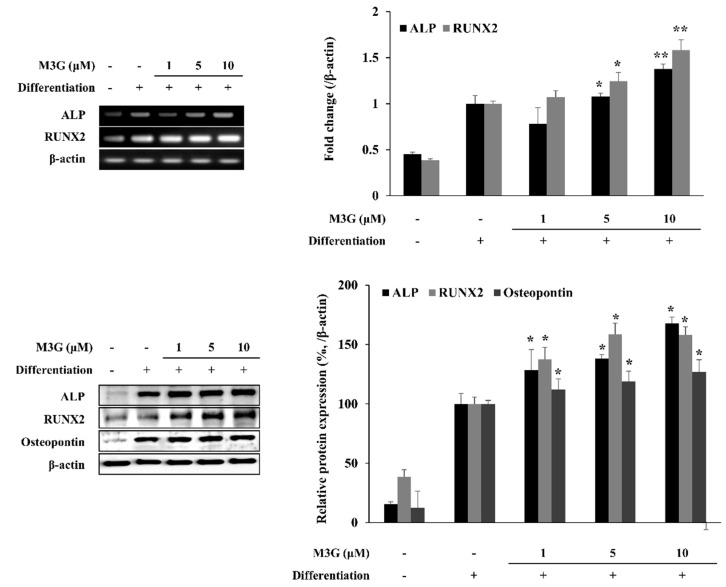
Effect of M3G on osteoblastogenesis marker gene expression. Analysis of gene expression was carried by measuring mRNA and protein levels in osteo-induced hBM-MSCs via RT-PCR and Western blot, respectively, at day 10 differentiation. Osteo-induced hBM-MSCs were treated with M3G until day 3 of differentiation. * *p* < 0.05, ** *p* < 0.01 vs. differentiated untreated group.

The effect of M3G on osteoblast marker genes and proteins was analyzed with RT-PCR and Western blotting, respectively, at day 10 differentiation in osteo-induced hBM-MSCs. The mRNA expression levels of ALP and RUNX2 as osteoblastogenesis markers increased with the induction of osteoblastogenesis in untreated hBM-MSCs. The presence of M3G dose-dependently increased the mRNA expression of ALP and RUNX2 compared to the untreated differentiated control group. Similar results were obtained from Western blotting. Protein levels of ALP and RUNX2 were increased by differentiation and further stimulated by M3G treatment. In addition, the osteopontin levels were also observed to be increased by M3G treatment (Figure 3).

### 3.3. Effect of M3G on Canonical Wnt/BMP Signaling during Osteoblastogenesis

**Figure 4:**
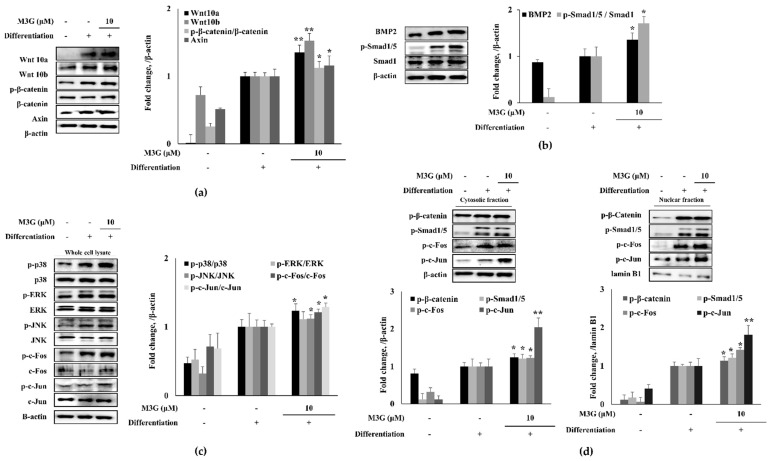
Effect of M3G on Wnt/β-catenin (**a**), BMP (**b**) and MAPK (**c**) signaling pathways and their downstream nuclear effectors (**d**). Analysis of protein expression was carried out by Western blotting of osteo-induced hBM-MSCs at day 10 differentiation. M3G was treated with initial differentiation induction (3 days) and it was not present in subsequent media changes. β-actin (for whole cell and cytosolic fraction) and lamin B1 (for nuclear fraction) were used as internal loading control. * *p* < 0.05, ** *p* < 0.01 vs. differentiated untreated group.

In order to elucidate the mechanism of action behind the osteoblastogenesis stimulation activity of M3G, the canonical Wnt signaling was investigated. The levels of signaling proteins and their phosphorylation were analyzed by Western blotting. The hBM-MSCs expressed significantly increased levels of Wnt10a and Wnt10b proteins following the inducement of osteoblastogenesis. Expectedly, phosphorylated β-catenin levels were observed along with Axin. The M3G (10 μM)-treated group exhibited increased levels of Wnt10a and Wnt10b compared to the untreated differentiated control group. In a similar trend, M3G treatment also increased the levels of β-catenin phosphorylation and free Axin protein levels (Figure 4a). Very similar results were obtained from the BMP signaling results. BMP2 and the phosphorylation of its downstream activator Smad1/5 complex were significantly increased during osteoblastogenesis, and with M3G treatment protein levels of BMP2 and phosphorylated Smad1/5 complex were further increased compared to untreated osteoblasts (Figure 4b). As a transcriptional activator signaling cascade, MAPK signaling was also investigated in osteo-induced hBM-MSCs. Phosphorylation of p38, ERK, and JNK MAPKs were all stimulated in osteoblasts (Figure 4c) and, similar to previous results, treatment with M3G further elevated the phosphorylated levels of p38 and JNK MAPKs but not ERK. In addition, as the downstream effectors of p38 and JNK, respectively, phosphorylation of c-Fos and c-Jun were similarly increased first by osteoblastogenesis inducement and further with M3G treatment. 

The Wnt and BMP signaling was further analyzed by investigating the nuclear and cytosolic levels of transcriptional activators: phosphorylated β-catenin, Smad1/5, c-Fos, and c-Jun. The nuclear levels of phosphorylated β-catenin, Smad1/5, c-Fos, and c-Jun were all elevated in osteoblasts and in a similar fashion to previous results, M3G presence elevated the nuclear levels of these transcription factors (Figure 4d).

**Figure 5:**
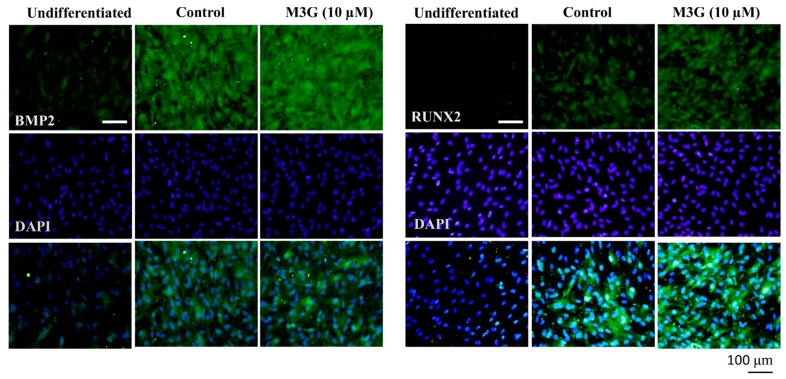
Effect of M3G on osteoblastogenesis marker protein expression. Analysis of protein expression was carried out by immunofluorescence staining of osteo-induced hBM-MSCs at day 10 differentiation. M3G was treated with initial differentiation induction (3 days) and M3G was not present in subsequent media changes. β-actin was used as internal loading control. Scale bar: 100 μm.

The effect of M3G on osteoblastogenesis was further examined by immunohistochemical staining of the BMP and RUNX2 proteins. Images of the cells showed that M3G treatment significantly stimulated the BMP and RUNX2 protein expression in osteo-induced hBM-MSCs (Figure 5).

### 3.4. Effect of M3G on the Lipid Accumulation of hBM-MSC Adipocytes

**Figure 6:**
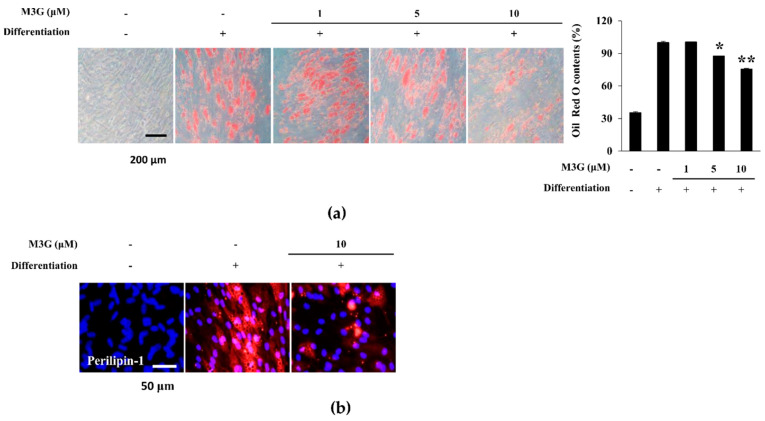
Effect of M3G on adipogenic differentiation of hBM-MSCs. (**a**) Effect of M3G on the intracellular lipid accumulation in adipo-induced hBM-MSCs at day 10 of differentiation. Lipid droplets were stained with Oil Red O and the quantification was carried out by measuring absorbance values of retained dye. Lipid accumulation level was given as relative percentage of Oil Red O dye compared to adipo-induced untreated group. * *p* < 0.05, ** *p* < 0.01 vs. differentiated untreated group. (**b**) Effect of M3G on the expression of perilipin-1 in adipo-induced hBM-MSCs at day 10 differentiation analyzed by immunofluorescence staining. DAPI staining was used to highlight the nucleus of viable cells. Scale bar: 50 μm. M3G was treated with initial differentiation induction (3 days) and was not present in subsequent media changes.

As a marker of adipocyte maturation, lipid accumulation of adipo-induced hBM-MSCs was investigated by staining the intracellular lipid droplets. At day 10 of differentiation, adipo-induced hBM-MSCs showed accumulated intracellular lipid droplets which were dose-dependently decreased by M3G treatment (Figure 6a). The effect of M3G on lipid accumulation is further confirmed by the immunohistochemical staining of perilipin-1, a protein known as lipid droplet-associated protein. Cell images clearly showed that the significant increase in perilipin-1 levels in hBM-MSC adipocytes was reverted by the presence of M3G (10 μM) (Figure 6b).

### 3.5. Effect of M3G on the Expression of Adipogenesis Marker Genes and Proteins

**Figure 7:**
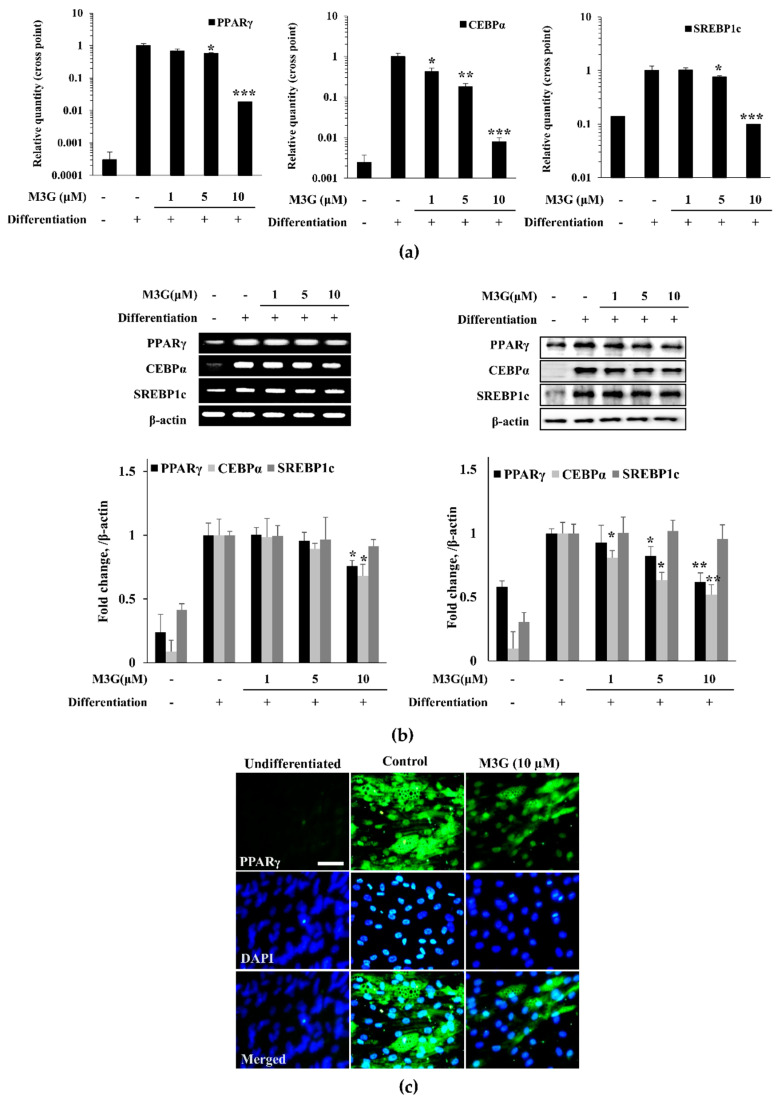
Effect of M3G on the expression of adipogenic marker genes (**a**) and proteins (**b**). Analysis of gene and protein expression of adipo-induced hBM-MSCs was carried out by RT-PCR and Western blot, respectively, at day 10 of differentiation. β-actin was used as internal loading controls. (**c**) Effect of M3G on the expression of PPARγ in adipo-induced hBM-MSCs at day 10 differentiation analyzed by immunofluorescence staining. DAPI staining was used to highlight the nucleus of viable cells. Scale bar: 50 μm. M3G was treated with initial differentiation for 3 days. * *p* < 0.05, ** *p* < 0.01, *** *p* < 0.001 vs. differentiated untreated group.

The effect of M3G on adipogenesis of hBM-MSCs was further investigated by the mRNA and protein expression of adipogenesis markers: PPARγ, CEBPα, and SREBP1c. The mRNA expression of adipogenic markers was analyzed by RT-qPCR. The expression of all three adipogenic markers was significantly increased by the inducement of adipogenesis (Figure 7). However, treatment with M3G suppressed the expression of PPARγ, CEBPα, and SREBP1c in a dose-dependent manner. Correlative results were obtained from the examination of protein levels. M3G treatment dose-dependently inhibited the protein levels of PPARγ and CEBPα which were stimulated in untreated hBM-MSC adipocytes. However, the effect of M3G on SREBP1c protein levels was not as significant as others (Figure 7b).

The effect of M3G on adipogenic marker genes was further confirmed by immunohistochemical staining of the adipo-induced hBM-MSCs for PPARγ. Cell images clearly showed that M3G treatment significantly decreased the PPARγ levels in adipo-induced hBM-MSCs (Figure 7c).

### 3.6. Effect of M3G on MAPK/AP-1 Signaling in Adipo-Induced hBM-MSCs

**Figure 8:**
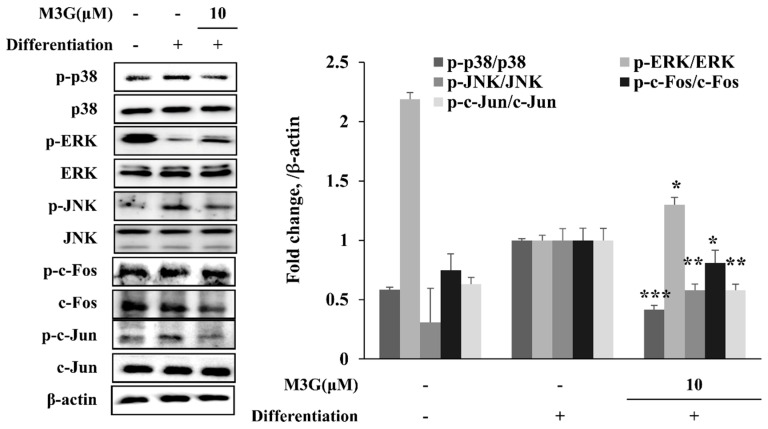
Effect of M3G on the MAPK/AP-1 signaling. Analysis of MAPK and AP-1 activation and was carried out with Western blotting of whole-cell lysates of adipo-induced hBM-MSCs at day 10 of differentiation. M3G was treated with initial differentiation for 3 days. β-actin was used as an internal loading control. * *p* < 0.05, ** *p* < 0.01, *** *p* < 0.001 vs. differentiated untreated group.

In order to evaluate the mechanism behind the effect of M3G on adipogenic differentiation of hBM-MSCs, the phosphorylation of MAPK/AP-1 signaling was examined. The hBM-MSCs induced for adipogenesis exhibited significantly increased phosphorylation of p38 and JNK where ERK phosphorylation was suppressed. M3G treatment reverted the effect of adipogenesis on MAPKs, suppressing the p38 and JNK phosphorylation while stimulating that of ERK. Similar results were obtained from the analysis of MAPK downstream activator c-Fos and c-Jun. Adipo-induced hBM-MSCs exhibited suppressed phosphorylation of c-Fos and c-Jun under M3G treatment which was otherwise significantly elevated (Figure 8).

## 4. Discussion

Currently, various plant-origin phenolic compounds are being studied and reports show that these compounds are potent nutraceuticals with promising health beneficial effects varying from treating symptoms of several diseases to the prevention of metabolic disorders such as diabetes, obesity, and osteoporosis [5,6]. Plant-based nutraceuticals can be found in common dietary sources in various forms and derivatives which may show different and/or more efficient bioactivities. Myricetin is a common phytochemical which is intensively studied for its bioactivities and still provides novel outcomes in terms of efficiency and effectiveness [10,17,18]. Therefore, derivatives of myricetin are also receiving increasing interest in this context where their potential bioactivities, action mechanisms, adverse effects, etc., are being continuously explored. On account of myricetin being a promising compound with reported activities against obesity and related complications, this study focused on the evaluation of a myricetin glycoside, M3G, for its effect on adipogenic and osteogenic differentiation of hBM-MSCs to obtain data regarding its anti-osteoporotic potential.

Reports showed that myricetin has beneficial effects on osteogenic differentiation, bone formation, and bone repair, while also being an anti-obesity agent with adipogenic inhibitory properties [19,20,21]. Myricetin was shown to induce osteoblast differentiation of different origin cell lines via similar mechanisms of BMP-2 and MAPK activation [22,23]. However, the effect of myricetin was also observed to be dependent on the treatment time, treatment period, and the cell line origin. The studies showed that during the onset of osteoporosis, the commitment of the MSCs in favor of adipogenesis occurs at the early stages of differentiation [24] where antagonistic feedback between PPAR and Wnt signaling takes place to suppress Wnt-mediated osteogenic induction while enhancing PPAR expression [25]. Therefore, the current study aimed to analyze the effect of M3G on the early stages of MSC commitment, after the introduction of differentiation medium (osteogenic or adipogenic). The current results reported a similar effect for the M3G when treated only during the initial differentiation inducement (first 3 days). This result suggested that the osteoblast differentiation enhancement effect of M3G appeared through its intervention on the early osteoblastogenesis signaling such as the activation of the transcriptional activities of RUNX2 and canonical Wnt signaling. Results confirmed this as the enhancement of mRNA and protein levels of RUNX2 in differentiated hBM-MSCs in the presence of M3G up to 10 µM. It was also accompanied by the elevation of osteopontin which is a protein expressed as a downstream product of RUNX2 activation. The role of both canonical and non-canonical Wnt signaling and its intertwined relationship with PPAR pathways during osteoporosis have been reported [24]. Additionally, He and Su [25] showed that the antagonist relationship between Wnt and PPAR pathways has regulatory roles in a dexamethasone-induced osteoporosis model. They suggested that the enhanced expression of PPAR by dexamethasone subsequently suppressed osteogenic differentiation which underlies glucocorticoid-induced osteoporosis.

Therefore, Wnt signaling was investigated as the upstream activator of osteoblastogenesis. This canonical pathway regulates osteoblast differentiation via RUNX2. Treatment of the osteo-induced hBM-MSCs with M3G resulted in elevated levels of Wnt10a and Wnt10b levels which are osteoblast differentiation-associated ligands expressed highly to activate the Wnt signaling [26]. Activation of Wnt signaling induces the stabilization and accumulation of β-catenin protein and its consecutive nuclear translocation. Current results showed that following M3G treatment the levels of nuclear β-catenin significantly increased indicating that M3G treatment enhanced the Wnt activation. Similar results were obtained from the BMP-2 signaling cascade. BMP-2 signaling is a mid and late osteoblast differentiation pathway which is activated by canonical Wnt signaling and responsible for the osteoblast maturation and consequent bone formation [27,28]. M3G treatment significantly enhanced BMP-2 expression and the phosphorylation of its downstream activator Smad1/5 complex compared to untreated differentiated hBM-MSC osteoblasts. In a similar manner to that of Wnt signaling, treatment with M3G also increased the nuclear levels of phosphorylated Smad1/5 complex. Overall, results indicated that M3G presence had beneficial effects on the activation of Wnt and BMP signaling pathways during osteoblast differentiation of hBM-MSCs. 

Reports indicated that Wnt10a and Wnt10b also had an antagonistic relationship with adipogenic differentiation of MSCs [29] where activation of Wnt signaling inhibits the adipogenic signaling cascade while inducing osteogenic differentiation. Considering that increased adipogenic differentiation of bone marrow MSCs as opposed to osteogenic differentiation is one of the reasons behind porous bones seen in osteoporosis [30], the effect of M3G on adipogenic differentiation of hBM-MSCs was also investigated. Reports showed that activation of Wnt signaling during the early stages of osteoblast differentiation suppresses PPARγ-mediated inducement of adipogenic signaling. First, the effect of M3G on the lipid accumulation of adipo-induced hBM-MSCs showed that M3G had a potential inhibitory effect on the differentiation of adipocytes. This was confirmed with the M3G-mediated suppression of PPARγ mRNA and protein expression levels compared to untreated differentiation hBM-MSC adipocytes. Along with PPARγ, M3G treatment also suppressed the expression of downstream adipogenic transcription factors SREBP1c and C/EBPα [31]. Some studies reported that the regulation of PPARγ gene expression is partly controlled through the MAPK activation and transcriptional activities of its downstream transcription factor AP-1 [32,33]. AP-1 is a complex formed by phosphorylated c-Fos and c-Jun, two proteins activated by MAPK signaling. Therefore, the effect of M3G on MAPK signaling was also examined to confirm its role in suppressing PPARγ-mediated adipogenesis. The results showed that the phosphorylation of p38 and JNK MAPKs were significantly increased in adipocytes while the M3G treatment resulted in decreased levels of p38 and JNK phosphorylation. On the other hand, ERK1/2 phosphorylation was suppressed in hBM-MSC adipocytes at day 10 differentiation. Moreover, M3G treatment relieved the adipogenic suppression of ERK1/2. Although ERK activation was reported to be a part of adipogenesis at early stages, it was also shown that mature adipocytes exhibited suppressed ERK1/2 activation [34].

In the current study, adipo-inducement of hBM-MSCs resulted in increased phosphorylation of p38 and JNK MAPKs and reduced ERK phosphorylation. This was suggested to be due to negative regulation of the adipogenic maturation during later stages of adipogenesis via ERK1/2 activation [34]. Overall, treatment with M3G significantly suppressed the adipogenesis in induced hBM-MSCs through the PPARγ pathway. This is in agreement with previous reports where myricetin and other polyphenols with similar structures such as quercetin and kaempferol inhibited the early stages of adipogenesis via suggested interaction with PPARγ [35,36]. Furthermore, the antagonistic relationship between Wnt and PPARγ signaling [37] might play role in the adipogenesis inhibitory effect of M3G in hBM-MSCs. The therapeutic potential of the Wnt/β-catenin pathway and tilting the differential tendencies of MSCs towards osteoblast while suppressing their adipogenesis as a target for osteoporosis treatment have been reported [37,38]. Shifting the differentiation balance of bone marrow stromal cells in favor of osteoblastogenesis had attenuative effects on osteoporosis-mediated damages in bone tissue. Considering all, it was suggested that M3G enhanced osteoblastogenesis while suppressing adipogenesis in hBM-MSCs through Wnt and PPARγ pathways, respectively. 

Prior to the results of the current study, the potential of myricetin, of which M3G is derived, has been shown in vivo with several disease models. Fan et al. [39] showed that myricetin supplement alleviated dexamethasone-induced osteoporosis in Sprague-Dawley rats through the promotion of osteogenic differentiation. Similar to M3G, myricetin exerted its effects via ERK signaling pathway in the aforementioned study. Additionally, in a study by Ying et al. [20], myricetin also exhibited osteoprotective effects in diabetic rats as well. The anti-adipogenic effects of myricetin were shown in a study by Su et al. [40] where myricetin supplement protected C57BL/6 mice from diet-induced obesity via PPARγ-linked signaling pathways. Angelicin, a natural bioactive compound, was also shown to prevent osteoporosis in ovariectomized rat models by regulating Wnt and PPAR signaling which suggests a potential role against postmenopausal osteoporosis [41]. Parallel to reported in vivo activities of myricetin, M3G was shown to interact with similar pathways in vitro to promote osteoblastogenesis and inhibit adipogenesis.

In conclusion, M3G was shown to be a potential bioactive compound with potential beneficial effects on bone structure. It was hypothesized that M3G may exhibit its activities via enhancing differentiation of bone marrow mesenchymal stromal cells into osteoblasts while hindering adipocyte differentiation to stimulate bone formation. The results suggested M3G as a potential lead natural product as a derivative of myricetin to develop anti-osteoporotic nutraceuticals, although further in vivo and detailed mechanism of action studies are urged for the proper utilization of M3G.

## Data Availability

All data used to support the findings of this study are available from the corresponding author upon reasonable request.
